# Benign Phyllodes Tumor With Cystic Squamous Metaplasia: A Cytohistological Correlation of a Rare Case

**Published:** 2017-07-01

**Authors:** Indranil Chakrabarti, Priyanka Agarwala, Pranati Bera, Sankarsan Bhaduri

**Affiliations:** 1 *Dept. of Pathology, North Bengal Medical College, The West Bengal University of Health Sciences, Siliguri, Darjeeling, West Bengal, India*; 2 *Anadaloke Sonoscan Centre, Siliguri, Darjeeling, West Bengal, India*

**Keywords:** Benign Phyllodes Tumor, Cystic Squamous Metaplasia, Cytology

## Abstract

**Background:**

Phyllodes tumors (PTs) are uncommon biphasic fibroepithelial neoplasms of the breast occurring in elderly females with a peak incidence between 45 and 49 years. Depending on various histological criteria, they are subdivided into benign, borderline, and malignant forms. Metaplastic changes occur, but are quite infrequent and cystic squamous metaplasia is very rare among the observed metaplastic changes in PT.

**Case:**

The current paper presents the case of a 41-year-old female with a progressively enlarging swelling in the left breast. Subsequent histopathological examination revealed benign PT with cystic squamous metaplasia. The previously done fine needle aspiration smears were also reviewed, which showed important diagnostic clues to this rare entity, but were ignored due to the rarity of the lesion.

**Conclusion:**

The case was presented because of its unique cytological and histopathological morphology and also to determine the role of aspiration cytology to diagnose such a rare occurrence.

## Introduction

Phyllodes tumors (PTs) are a group of circumscribed biphasic fibroepithelial tumors characterized by a double layered epithelial component surrounded by an overgrowing hypercellular mesenchymal component organized in leaf-like structure. ^[1]^ These are uncommon tumors accounting 0.3% to 1% of all primary breast tumors (1), with the overall incidence of 2.1/million (2). The peak age of incidence is 40 to 50 years overall (1), whereas in Asian countries it occurs in younger ages (average 25 to 30 years) (3). PT can be subclassified histologically as benign, borderline, and malignant according to features such as tumor margins, stromal overgrowth, necrosis, cellular atypia, and the number of mitoses per high-power field (4). Metaplastic changes can be observed in PT in epithelial or stromal components. The stroma may show cartilaginous, osteoid, or lipomatous metaplasia, while epithelium may occasionally show apocrine or squamous metaplasia (5), of which cystic squamous metaplasia is very rare, accounting only 10% of PT, making PT with cystic squamous metaplasia an extremely rare primary breast tumor (6,7).

## Case report

A 41-year-old female referred to the surgical outpatient department with chief complaints of swelling in the left breast of five years’ duration, which rapidly increasing in size for the last four months with no history of trauma or any constitutional symptoms.

Her general and systemic examination was within normal limit. Local examination showed a well-defined irregular mass of 9 cm in diameter approximately, not fixed to the skin or chest wall, firm in consistency, but soft at places and tender on palpation. Ultrasonograhic evaluation revealed an 8.9 x 6.6 x 3.9 cm heterogeneous soft tissue mass in retroareolar and periareolar regions of the left breast.

The mass was excised and sent to the Department of Pathology, North Bengal Medical College, The West Bengal University of Health sciences, India for histopathological examination.

**Gross examination**

**Figure 1:**
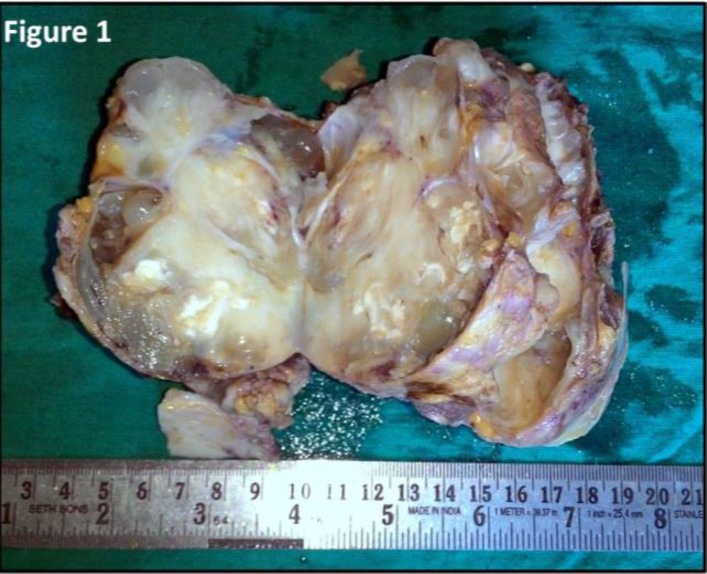
Gross examination of cut section showing mucoid areas and cystic spaces with whitish material within the solid tumor mass

Gross examination showed a mass with attached overlying skin measuring 10 x 7 x 6 cm. Serial cut sections revealed multiple cystic spaces with mucoid material and few cysts containing whitish material and well defined whitish homogenous areas with one area showing leaf-like mass projecting inside a cystic cavity (Figure 1).

**Microscopic examination**

**Figure 2:**
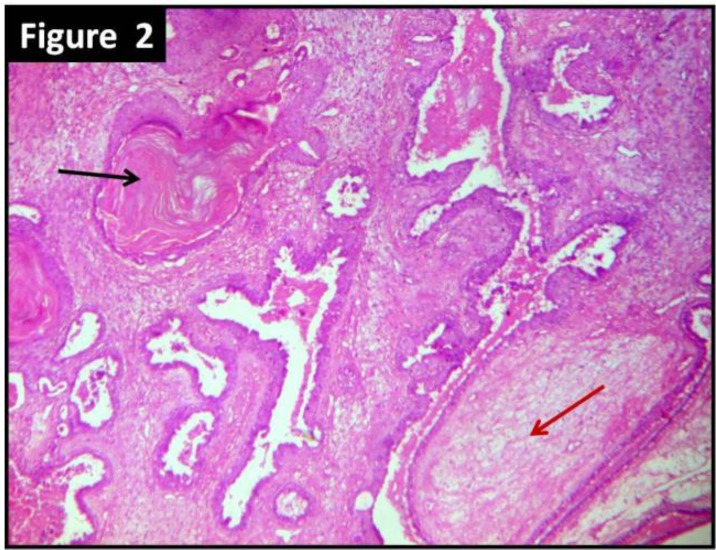
Photomicrograph showing benign phyllodes tumor (red arrow) with squamous epithelium lined cystic spaces with lamellated keratin (black arrow) (hematoxylin and eosin stain; X100 magnification)

**Figure 3:**
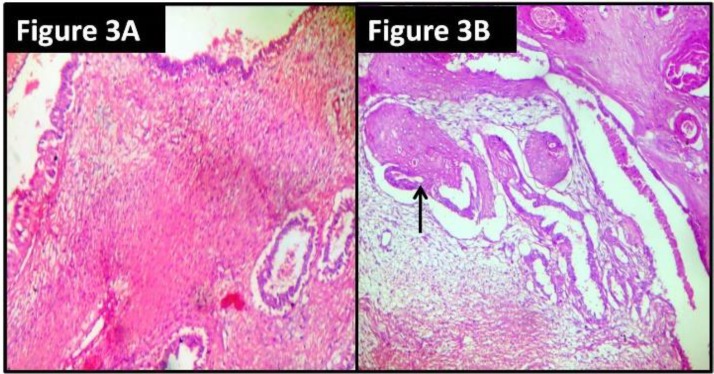
Photomicrograph showing stromal hypercellularity (Figure 3A) and ductal epithelium showing squamous metaplasia (Figure 3B) (hematoxylin and eosin stain; X100 magnification)

**Figure 4:**
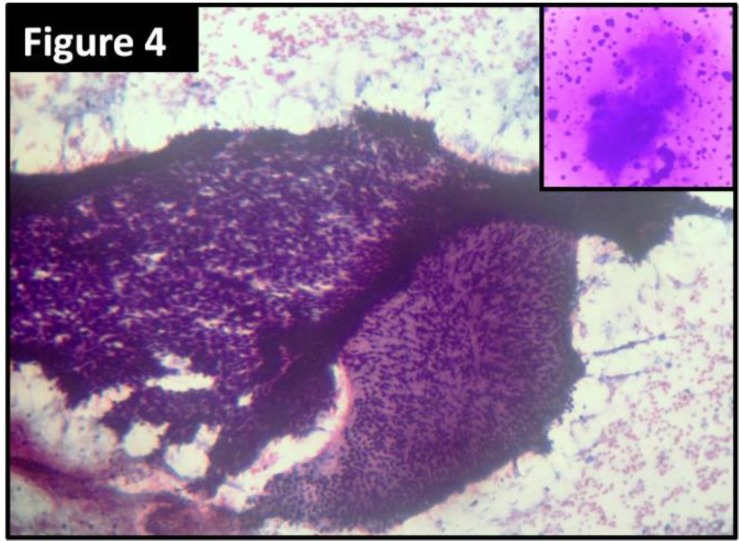
Photomicrograph from cytological smears show hypercellular fragments of benign phyllodes tumor (hematoxylin and eosin stain; X100 magnification) Inset shows keratinous material (Leishman stain)

Multiple sections were taken from representative areas, processed, and examined. The periphery of the tumor was well-circumscribed. The sections showed features of benign PT characterized by stromal hypercellularity and stromal overgrowth. There was no cellular atypia and mitotic activity was low. The sections also revealed multiple cystic and glandular spaces enclosed within cellular stroma. The spaces were lined by multilayered benign squamous cells and the cystic spaces were filled with keratin material (figures 2 and 3). Preoperative fine–needle aspiration cytology (FNAC) was performed in a private diagnostic centre. The slides were reviewed and they showed features suggestive of benign phyllodes tumor with cystic squamous metaplasia, which was consistent with the histopathological findings (Figure 4).

## Discussion

PTs, uncommon biphasic fibroepithelial neoplasms (8), usually occur in females aged 45 to 49 years (9,10), but can also occur in younger adults and adolescents (11). World Health Organization (WHO) classified PT into benign, borderline, and malignant, based on histological characteristics that include degree of stromal cellular atypia, degree of stromal overgrowth, mitotic activity per 10 high power fields, tumor necrosis, and margin appearance (the first 3 being the main parameters) (12). Metaplastic changes are infrequent in the stromal and epithelial components of PT (13). In one of the reported PT series, 11 out of 335 cases (3.3%) showed malignant heterologous components such as osteosarcoma, liposarcoma, and rhabdomyosarcoma. The epithelial component infrequently showed usual-type epithelial hyperplasia, epithelial squamous metaplasia observed in 12 cases (3.6%), and apocrine metaplasia. Five out of 12 cases with squamous metaplasia revealed squamous cysts. FNAC from cystic areas of tumor may lead to a mistaken diagnosis of squamous cyst (13). Other than PTs, cases of extensive squamous metaplasia in the epithelium of gynecomastia and benign breast papillomatosis were reported (14,15). Differential diagnosis of PT includes fibroadenoma with cellular stroma, adenoma, hamartoma, lipoma, juvenile papillomatosis, carcinoma, sarcomas, and metastasis (12). Histologically, fibroadenomatous stroma is usually homogenous, and it is important to distinguish it from PTs, which can exhibit considerable stromal heterogeneity (16). It is suggested that the development of squamous metaplasia in the breast begins within the myoepithelial cell layer, as supported by immunohistochemical studies (by expression of actin, vimentin, and S-100 protein in the metaplastic squamous cells) (13).

## Conclusion

Hence, the current case was presented due to its unique cytological and histopathological morphology and also to determine the role of aspiration cytology to diagnose such a rare occurrence.
